# Ophthalmoplegia Due to Miller Fisher Syndrome in a Patient With Myasthenia Gravis

**DOI:** 10.3389/fneur.2019.00823

**Published:** 2019-08-13

**Authors:** Roberta Brusa, Irene Faravelli, Delia Gagliardi, Francesca Magri, Filippo Cogiamanian, Domenica Saccomanno, Claudia Cinnante, Eleonora Mauri, Elena Abati, Nereo Bresolin, Stefania Corti, Giacomo Pietro Comi

**Affiliations:** ^1^Neurology Unit, Department of Pathophysiology and Transplantation, Dino Ferrari Center, IRCCS Foundation Ca' Granda Ospedale Maggiore Policlinico, Milan, Italy; ^2^Neurology Unit, Department of Pathophysiology and Transplantation, Dino Ferrari Center, IRCCS Foundation Ca' Granda Ospedale Maggiore Policlinico, University of Milan, Milan, Italy; ^3^Neuropathophysiology Unit, IRCCS Foundation Ca' Granda Ospedale Maggiore Policlinico, Milan, Italy; ^4^Neuroradiology Unit, IRCCS Foundation Ca' Granda Ospedale Maggiore Policlinico, Milan, Italy

**Keywords:** myasthenia gravis, Miller Fisher syndrome, autoimmune diseases, ophthalmoplegia, GQ1b

## Abstract

Here, we describe a 79-year-old man, admitted to our unit for worsening diplopia and fatigue, started a few weeks after an episode of bronchitis and flu vaccination. Past medical history includes myasthenia gravis (MG), well-controlled by Pyridostigmine, Azathioprine, and Prednisone. During the first days, the patient developed progressive ocular movement abnormalities up to complete external ophthalmoplegia, severe limb and gait ataxia, and mild dysarthria. Deep tendon reflexes were absent in lower limbs. Since not all the symptoms were explainable with the previous diagnosis of myasthenia gravis, other etiologies were investigated. Brain MRI and cerebrospinal fluid analysis were normal. Electromyography showed a pattern of predominantly sensory multiple radiculoneuritis. Suspecting Miller Fisher syndrome (MFS), the patient was treated with plasmapheresis with subsequent clinical improvement. Antibodies against GQ1b turned out to be positive. MFS is an immune-mediated neuropathy presenting with ophthalmoplegia, ataxia, and areflexia. Even if only a few cases of MFS overlapping with MG have been described so far, the coexistence of two different autoimmune disorders can occur. It is always important to evaluate possible differential diagnosis even in case of known compatible diseases, especially when some clinical features seem atypical.

## Background

Myasthenia gravis (MG) is an autoimmune disorder of the neuromuscular junction characterized by fatigability and fluctuating weakness of voluntary muscles, leading to symptoms such as diplopia, palpebral ptosis, dysphagia, dyspnea, or limb weakness. Disease exacerbations can be triggered by concomitant infections, vaccination, or administration of certain antibiotics. Antibodies against acetylcholine receptor (AchR), muscle-specific kinase (MuSK), and low-density lipoprotein receptor-related protein 4 (LRP4) may be found on serum. Treatment generally involves symptomatic therapy with acetylcholinesterase inhibitors and immunosuppression, despite initiation of high doses of steroids can precipitate symptoms.

## Case Presentation

A 79-year-old man was admitted to our emergency room complaining fatigue, generalized weakness, nausea, and worsening of usual diplopia, following an episode of bronchitis treated with cephalosporins. Moreover, he received flu vaccination a few weeks before the onset.

MG was diagnosed 8 years earlier after the onset of lid ptosis and head drop (class IIA according to the MGFA classification). Antibodies against acetylcholine receptor (AChR) and ryanodine receptor (RyR) were detected, but chest CT scan was negative for thymoma. So far, clinical symptoms had always been well-controlled by pyridostigmine, azathioprine, and low doses of steroids. Last neurological examination, performed 2 months earlier, revealed only mild diplopia on left lateral gaze after prolonged fixation.

The patient also suffered from a mild iatrogenic chronic sensorimotor axonal polyneuropathy due to chemotherapy administered after surgical resection of colon cancer; he also underwent ablation of two metastatic lesions in the liver, but the subsequent oncological follow-up was reported negative.

On admission, neurological examination revealed diplopia on lateral gaze, limitation in upward gaze, mild ptosis of the left eye after prolonged fixation, uncertainties at finger-to-nose test, mild proximal limb muscles fatigability, and absence of knee and Achilles reflexes. Brain CT scan and chest X-rays were negative.

A putative diagnosis of worsening of myasthenia gravis was made and the patient was hospitalized due to concerns of respiratory failure, considering the age and the recent infection. Absence of lower limbs deep tendon reflexes was initially ascribed to the polyneuropathy. On admission, taking into account the mild presenting symptoms and the absence of respiratory involvement, Prednisone and Azathioprine dosages were increased (respectively from 7.5 to 12.5 and from 50 to 75 mg), withholding standard treatments for MG exacerbation, such as intravenous immunoglobulin or plasmapheresis.

In the first days of hospitalization, the patient developed progressive worsening of ocular movement abnormalities with horizontal and vertical gaze limitation, mild fluctuating diplopia in primary and lateral gaze, nystagmus on left lateral gaze, limb ataxia, and wide-based gait with multi-directional sways during Romberg maneuver. Additional findings included partial ptosis of the left eye, hands tingling, mild proximal weakness in lower limb, dysarthria, and facial asymmetry; nausea and episodes of vomiting persisted. While ophthalmoparesis could be compatible with MG, other clinical features suggested a different etiology.

Despite the fact that one of the most common presentations of MG is represented by ocular symptoms, acute bilateral ophthalmoparesis can be also attributed to various etiologies (Table 1).

**Table 1:** Causes of acute bilateral ophthalmoparesis.

*Brainstem*
Stroke
Hemorrhage
Tumor
Multiple sclerosis
Wernicke encephalopathy
*Cranial nerves*
Diabetic or vascular complications
Tuberculous meningitis and other infections
Guillain-Barrè or Miller-Fisher syndrome
*Cavernous sinus*
*Neuromuscular junction*
Botulism
Myasthenia gravis
*Muscles*
Myositis
Mitochondriopathies
*Orbit*
Cellulitis
Graves disease

Blood tests were unremarkable, except for mild hypercholesterolemia and increase of inflammatory indexes (C-reactive protein 0.71 mg/dl—normal value <0.5 mg/dl; erythrocyte sedimentation rate 35—normal value <20). Thyroid function, serum folic acid, and vitamin B12 dosages were normal. Antibodies against AChR and GQ1b were dosed.

Electromyography, performed after 5 days from the symptom onset, revealed mildly increased latency of the compound muscle action potential (CMAP) of the right facial nerve and moderate chronic neurogenic abnormalities in the muscles innervated by the right L5 and the right C5–C6 nerve roots; right tibial and right ulnar nerve F-wave latency was normal. Repetitive nerve stimulation did not show a decremental response.

**Figure 1:**
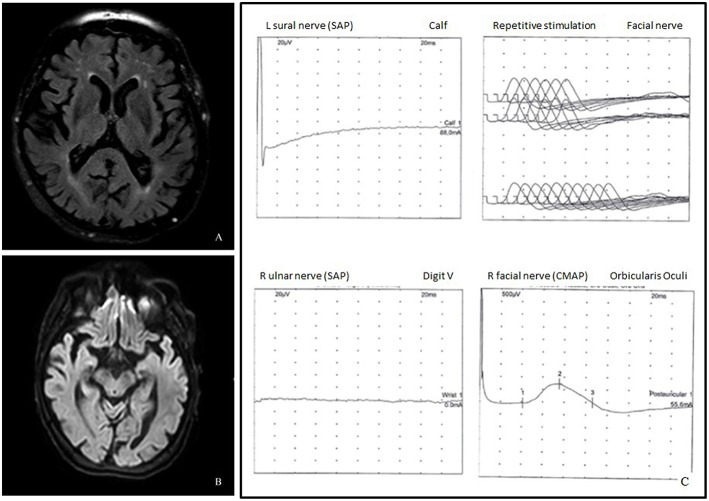
**(A)** Axial fluid attenuated inversion recovery (FLAIR) image demonstrated cortico-subcortical atrophy and chronic cerebrovasculopathy. **(B)** Diffusion weighted imaging (DWI) did not show acute lesions. **(C)** Neurophysiological studies showed absence of the left sural and the right ulnar sensory nerve action potentials, mildly increased latency of the facial nerve CMAP, and normal repetitive facial nerve stimulation.

Brain MRI revealed only mild chronic cerebrovascular disease and mild cortical and subcortical atrophy, without signs of acute cerebral lesions or Wernicke's encephalopathy (Figures 1A,B).

Clinical symptoms worsened up to complete external ophthalmoplegia and inability to walk unsupported with almost complete disappearance of upper limb reflexes. Another electromyography was repeated 3 days later, displaying a predominantly sensory polyradiculoneuropathy, characterized by increased facial nerve latency and absence of sural, ulnar, and radial sensory nerve action potentials (SNAP) (Figure 1C); ulnar nerve F-wave seemed not clearly detectable.

Cerebrospinal fluid (CSF) analysis, including virological investigations, was performed 6 days after symptom onset and resulted unremarkable, without albuminocytological dissociation (cells <1/mmc, proteins 24 mg/dl). Serum and CSF oligoclonal banding were absent.

Oncological screening, including neoplastic markers and full-body CT scan with contrast, performed due to the medical history of the subject, was negative for malignancies.

The workup suggested a diagnosis of acute polyradiculoneuropathy; thus, the patient was treated with five sessions of plasmapheresis, leading to stabilization of the clinical features and subsequent slow mild clinical improvement, especially of limb ataxia and, at a lesser extent, of gait ataxia and diplopia. Nausea and episodes of vomiting decreased too.

Antibodies against ganglioside GQ1b turned out to be positive at high titer on serum (IgM 1/2560 and IgG 1/5120; cut-off <1/640), confirming the diagnosis of Miller-Fisher syndrome (MFS). Antibodies against AChR were positive but lower compared to the last known titer (36.3 pmol/ml → 7.2 pmol/ml; normal values < 0.5 pmol/ml).

At discharge, the patient still presented severe ophthalmoparesis (only minimal ocular movements were possible), exhaustible left palpebral ptosis, facial asymmetry, gait ataxia (need for external support, maintenance of Romberg with multi-directional swinging with closed eyes), and slight weakness in proximal lower limb muscles, and brachioradialis reflex reappeared, though greatly reduced.

The patient underwent a cycle of physiotherapy and rehabilitation. After 2 months, without further therapy modifications, he was able to walk independently and the ophthalmoparesis markedly improved. Clinical evaluation showed mild convergence strabismus in primary position, mild limitation in horizontal and upward gaze, diplopia after 15 s on left lateral gaze, mild ptosis in the left eye after exercise, presence of the biceps and patellar reflexes, absence of limb or gait ataxia. Last electromyography showed a slightly prolonged ulnar F wave and persistence of the axonal sensory polyneuropathy.

## Discussion

Miller Fisher syndrome (MFS), a rare variant of Guillain-Barrè syndrome (GBS), is an immune-mediated neuropathy presenting with (1) ophthalmoparesis, (2) ataxia, and (3) areflexia. Disease onset usually follows an upper respiratory tract or a gastrointestinal infection. The most common presenting symptom of MFS is diplopia due to bilateral external ophthalmoplegia; ataxia is often severe but areflexia is not always present; patients may also exhibit ptosis, pupillary abnormalities (anisocoria, sluggish direct response to light, light-near dissociation), mild limb weakness, facial nerve involvement (~30%), and autonomic dysfunction (~10%). Antibodies anti GQ1b (IgG) can be detected in the majority of the patients (around 90%), but they might also be found in patients with GBS with ophthalmoplegia, Bickerstaff's brainstem encephalitis, acute ophthalmoparesis without ataxia, or ataxia without ophthalmoparesis. Cytoalbuminologic dissociation is present only in half of the patients. The clinical course of MFS is generally self-limiting and resolves in weeks to months with an excellent outcome; treatment with intravenous immunoglobulin and plasmapheresis has been proposed, but their usefulness in improving the clinical outcome is still not well-established, except in cases of overlap with other subtypes of GBS (1, 2).

Neurophysiological studies may be normal: sensory nerve conduction studies may present abnormalities (reduced SAP amplitude) in some patients (~30%) and ~15% of subjects have an overlap of axonal GBS. Motor nerve conduction studies and F-wave latencies are generally normal. In our patient, the history of polyneuropathy and possibly overlap with GBS may explain the neurophysiological findings, even if MFS cases with prolonged F-wave latencies and reduced CMAP amplitudes have been described in the literature (3).

While at first the patient's symptoms could mimic an exacerbation of myasthenia gravis, he later developed the classical symptomatic triad of MFS and antibodies anti GQ1b confirmed the diagnosis. Despite several autoimmune diseases can sometimes overlap between each other, only four cases of MFS associated with MG have been described so far and have been treated with IVIG or plasmapheresis (4–7). Even in our subject, plasmapheresis was effective in limiting the progression of symptoms.

It could be speculated that the underlying dysregulation of the immune system leads to a predisposition to autoimmune disorders targeting the motor unit. A possible trigger could have been the upper respiratory tract infection, more typically described in MFS/GBS, but also a role of the vaccination cannot be ruled out since very rare cases of post-vaccinal MFS have been described (8).

Considering that MG and MFS are both infrequent immune-mediated disorders, the coexistence of these two diseases in the same patient represents a very peculiar finding. At first, this patient represented an even more difficult clinical challenge since both disorders might present with ophthalmoplegia. Alongside the description of a very rare co-occurrence of MG and MFS, this case provides useful considerations for everyday clinical practice. Indeed, a correct differential diagnosis is fundamental for appropriate medical management, given the different prognosis and evolution of distinct disorders. Therefore, distinguishing the proper etiology of symptoms must always remain a priority, especially in case of atypical or incomplete presentations, confounding factors and partially compatible pre-existing disorders.

## Ethics Statement

Written informed consent was obtained from the individual(s) for the publication of any potentially identifiable images or data included in this article.

## Author Contributions

RB contributed to the conception and drafting of the manuscript and acquisition of data. IF contributed to the drafting and revision of the manuscript and acquisition of data. DG, EM, EA, FM, NB, SC, and GC contributed to the revision of the manuscript. FC contributed to the performance of neurophysiological studies. DS contributed to the determination of the titer of GQ1b antibodies. CC contributed to the acquisition and analysis of radiologic data.

### Conflict of Interest Statement

The authors declare that the research was conducted in the absence of any commercial or financial relationships that could be construed as a potential conflict of interest.
